# Cutaneous Invasive Aspergillosis: Retrospective Multicenter Study of the French Invasive-Aspergillosis Registry and Literature Review

**DOI:** 10.1097/MD.0000000000001018

**Published:** 2015-07-02

**Authors:** Céline Bernardeschi, Francoise Foulet, Saskia Ingen-Housz-Oro, Nicolas Ortonne, Karine Sitbon, Gaëlle Quereux, Olivier Lortholary, Olivier Chosidow, Stéphane Bretagne, the French Mycosis Study Group

**Affiliations:** Dermatology Department, UPEC (CB, SI-H-O, OC); Mycology and Parasitology Department (FF); Pathology Department, AP-HP, Henri Mondor Hospital, Créteil (NO); Pasteur Institute, National Reference Center for Invasive Mycoses and Antifungals, Paris (KS, OL, SB); Skin Cancer Unit, Nantes University Hospital (GQ); Infectious Diseases and Tropical Medicine Department, AP-HP, Necker-Enfants malades Hospital, Necker-Pasteur Infectious Diseases Center, IHU Imagine, Paris (OL); Université Paris Descartes (NO, OL); Université Paris-Est-Créteil UPEC, Créteil (OC); Mycology and Parasitology Department, AP-HP, Saint Louis Hospital, Paris (SB); Université Paris Diderot (SB); INSERM Centre d’Investigation Clinique, Créteil, (OC); and EA EpidermE, UPEC, Créteil, France (SI-H-O, OC).

## Abstract

Invasive aspergillosis (IA) has poor prognosis in immunocompromised patients. Skin manifestations, when present, should contribute to an early diagnosis. The authors aimed to provide prevalence data and a clinical and histologic description of cutaneous manifestations of primary cutaneous IA (PCIA) and secondary CIA (SCIA) in a unique clinical series of IA and present the results of an exhaustive literature review of CIA. Cases of proven and probable IA with cutaneous manifestations were retrospectively extracted from those registered between 2005 and 2010 in a prospective multicenter aspergillosis database held by the National Reference Center for Invasive Mycoses and Antifungals, Pasteur Institute, France. Patients were classified as having PCIA (i.e., CIA without extracutaneous manifestations) or SCIA (i.e., disseminated IA). Among the 1,410 patients with proven or probable IA, 15 had CIA (1.06%), 5 PCIA, and 10 SCIA. Hematological malignancies were the main underlying condition (12/15). Patients with PCIA presented infiltrated and/or suppurative lesions of various localizations not related to a catheter site (4/5), whereas SCIA was mainly characterized by disseminated papules and nodules but sometimes isolated nodules or cellulitis. Histologic data were available for 11 patients, and for 9, similar for PCIA and SCIA, showed a dense dermal polymorphic inflammatory infiltrate, with the epidermis altered in PCIA only. Periodic acid Schiff and Gomori-Grocott methenamine silver nitrate staining for all but 2 biopsies revealed hyphae compatible with *Aspergillus*. *Aspergillus flavus* was isolated in all cases of PCIA, with *Aspergillus fumigatus* being the most frequent species (6/10) in SCIA. Two out 5 PCIA cases were treated surgically. The 3-month survival rate was 100% and 30% for PCIA and SCIA, respectively. Our study is the largest adult series of CIA and provides complete clinical and histologic data for the disease. Primary cutaneous IA should be recognized early, and cases of extensive necrosis should be treated surgically; its prognosis markedly differs from that for SCIA. Any suppurative, necrotic, papulonodular, or infiltrated skin lesion in an immunocompromised patient should lead to immediate biopsy for histologic analysis and mycological skin direct examination and culture.

## INTRODUCTION

Invasive aspergillosis (IA) is an opportunistic fungal infection caused by *Aspergillus* spp. in immunocompromised patients, usually after inhalation of spores. Invasive aspergillosis has been defined by the European Organization for Research and Treatment of Cancer/Invasive Fungal Infections Cooperative Group and National Institute of Allergy and Infectious Diseases Mycoses Study Group (EORTC/MSG) and classified in 3 levels of probability: proven aspergillosis defined by histologic evidence of compatible hyphae and isolation of the fungus by culture from a normally sterile site; probable aspergillosis defined by the association of host (risk) factors together with clinical and mycological (direct or indirect test) criteria; and possible aspergillosis defined by the combination of clinical evidence and appropriate host factors without mycological support.^1^ The mortality rate of IA has been reported to be between 35% and 55% at 12 weeks in immunocompromised patients.^2–5^ Early diagnosis , however, can improve survival.^6^ Risk factors, diagnostic tools, prognostic factors, and treatment have been reviewed, with consensus.^7^ Little is known about cutaneous manifestations of IA because of the unknown prevalence of the involvement and the limited number of dedicated series.^8^

Cutaneous invasive aspergillosis (CIA) is commonly divided into primary and secondary lesions, primarily accounting for necrotic lesions that result from direct inoculation of the fungus at the injury site and secondary lesions resulting from the blood spreading of hyphae.^8^ Cutaneous invasive aspergillosis is commonly described as presenting with more or less pruritic papules, nodules, or plaques with secondary escharotic evolution. Despite several adult case reports and pediatric series,^9^ few large series of CIA, however, have been published, and the clinical and histologic spectrum of the disease remain underdescribed.

Therefore, we performed a retrospective analysis of proven and probable cases of IA with cutaneous manifestations collected in France between 2005 and 2010 by an active surveillance network. We aimed to: provide data on the prevalence of cutaneous manifestations with primary CIA (PCIA) and secondary CIA (SCIA) in a unique clinical series, give a clinical and histologic description of CIA, and present the results of an exhaustive literature review of CIA.

## METHODS

### Data Source

The prospective active Surveillance des Aspergilloses Invasives en France (SAIF) network is a French anonymous centralized database (National Reference Center for Invasive Mycoses and Antifungals, Institut Pasteur, Paris) relying on voluntary (as the declaration of IA cases is not mandatory) but systematic declaration of IA by mycologists from predefined university hospitals in France.^5^ On the standardized anonymous declaration, a web-based file, practitioners had to systematically mention the presence of skin involvement. All records with cutaneous involvement for January 2005 to July 2010 were extracted and analyzed.

### Selection of Patients

Proven and probable cases of IA were retained according to the EORTC/MSG definition criteria (criteria published in 2002 for the period 2005–2007^10^ and published in 2008 for 2008–2010).^1^ We first extracted from the database all cases with notified cutaneous involvement. Then, we systematically reviewed patient charts. We included patients with cutaneous lesions in accordance with previously published lesions suggestive of cutaneous aspergillosis^8^ or infectious dermatitis (ie, papules, nodules, necrosis, pustules, ulcerations, and/or suppuration), then classified patients as having proven or probable CIA as follows:

1. Proven CIA was defined by a skin biopsy showing histologic evidence of compatible hyphae and/or positive mycological culture of the skin biopsy.
2. Probable CIA was considered in patients with proven or probable IA and skin involvement clinically compatible with an infectious origin but no histologic features of fungal infection and no positive skin culture biopsy. To consider these patients as having probable CIA, a positive cutaneous mycological sample taken by swab, however, was needed.

The following data were recorded from files for patients with proven or probable CIA: age, sex, underlying disease, clinical features and localization of cutaneous lesions, time between cutaneous lesion appearance and the skin biopsy, fungal identification, treatment, and 3-month survival. Skin biopsies were retrospectively reviewed by a dermatopathologist of our team (NO) with blinding to the type of CIA (primary or secondary).

Two groups of patients were defined: those with PCIA (ie, presenting a unique skin lesion and no visceral involvement) and those with SCIA, characterized by the association of skin and visceral involvement or the presence of multiple cutaneous lesions (in 2 or more noncontiguous areas) suggestive of disseminated disease even in the absence of organ involvement.

The research complied with French law and the Declaration of Helsinki (as adopted in 2000), and was approved by the local institutional research board committee (CPP-Ile de France IX). Approval of the Commission Nationale de l’Informatique et des Libertés (CNIL) was obtained, ensuring that patient data were kept anonymous according to French regulations.

### Literature Review Search Strategy

We search MEDLINE via PubMed for articles published from January 1985 to July 2014 by using the key words “skin”, “cutaneous”, and “invasive aspergillosis”. The literature search yielded 720 citations. From these, we included only English language studies of skin involvement with IA and including more than 3 patients. This research was completed by an analysis of reviews of cutaneous IA for the purpose of the discussion.

## RESULTS

### Collection of Data

**FIGURE 1:**
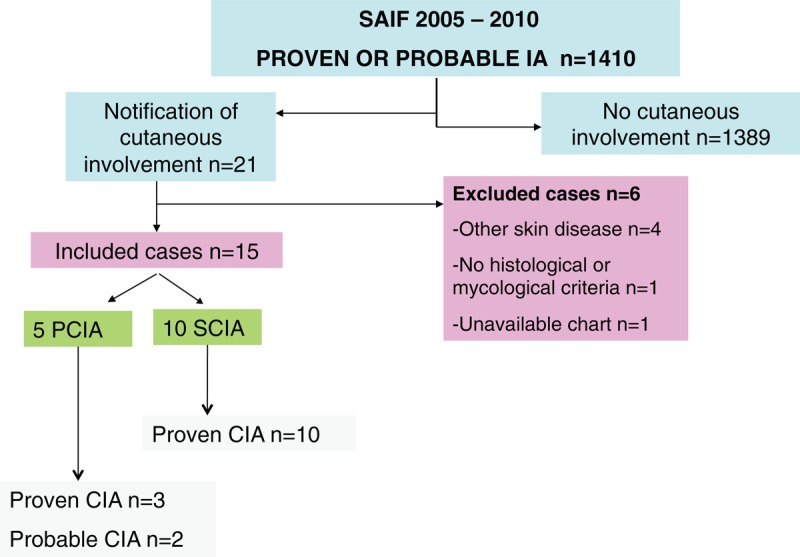
Flow chart of the study.

In total, 1410 cases of proven or probable IA were registered in the SAIF database, and 21 had cutaneous involvement noted (Figure 1). Six patients were excluded after systematic analysis of corresponding medical charts [cutaneous manifestations not consistent with cutaneous infection (drug reaction), N = 4; unavailable chart, N = 1; no histologic or mycological criteria, N = 1]. We included 15 patients, allowing to estimate to 1.06% the prevalence of skin lesions in proven or probable IA. According to our definitions, 13 patients presented proven CIA (skin histology findings, N = 9; positive mycological culture of skin biopsy, N = 13) and 2 probable CIA (positive swab culture). Five patients had PCIA and 10 had SCIA.

### Patient Characteristics

#### Epidemiological Data and General Conditions

The median age of patients was 56 years (range 1–90) and 9 (60%) were men; 12 (80%) had an underlying hematological malignancy and 4 (26%) a solid organ transplant (2 liver transplant, including a patient also with hematological malignancy, 1 heart, and 1 kidney transplant).

Visceral involvement in SCIA included the central nervous system (2/10), pulmonary (7/10), cardiac (1/10), and sinus (1/10). In 5 patients of PCIA, no systemic involvement was recorded.

Fever defined by temperature >38 °C was present in 5/10 SCIA and 5/5 PCIA cases. Septic shock was reported in 2/10 SCIA cases (1 because of *Escherichia coli* infection and 1 not documented) and 1/5 PCIA cases (not documented). In total, 1/5 patients with PCIA had an intravenous catheter adjacent to the fungal cutaneous lesion. Portal of entry was not found for other patients.

The median time between the appearance of the first cutaneous lesions and skin biopsy was 4 days (range 1–90 days) for SCIA and 5 days (range 0–19 days) for PCIA (Table 1 ).

**TABLE 1:**
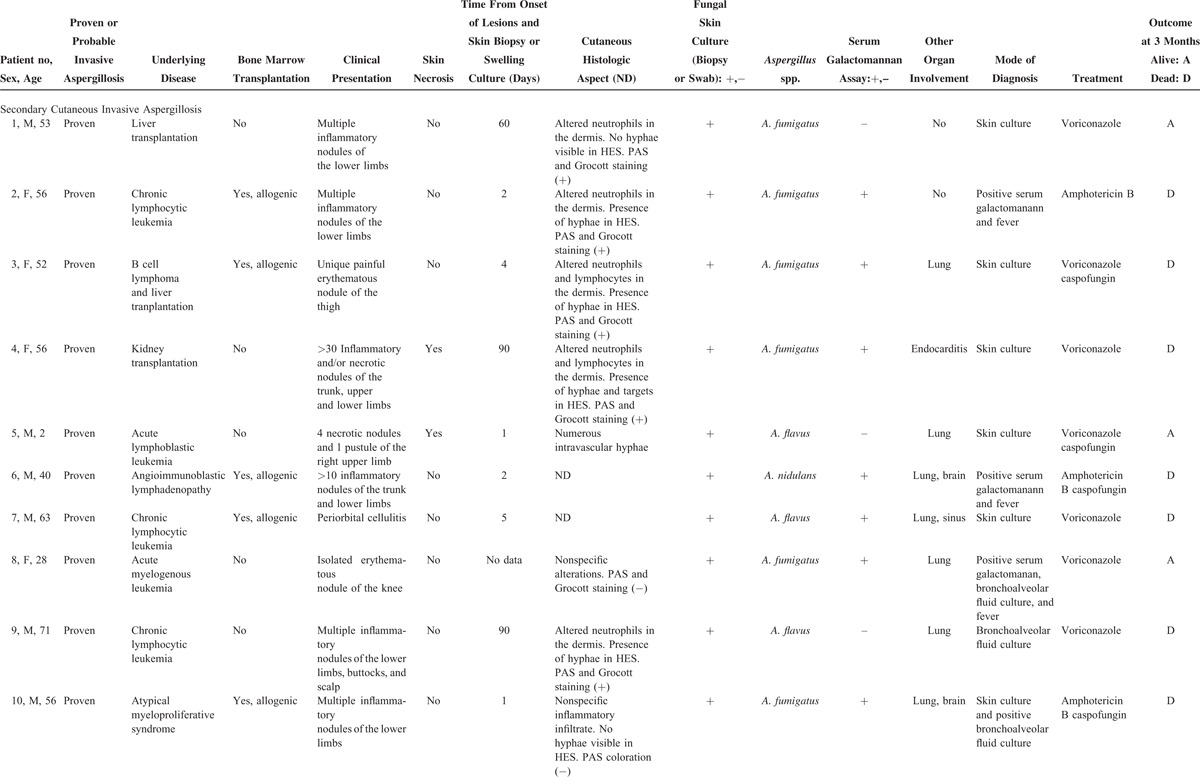
Clinical and Mycological Data for 15 Patients with Proven or Probable Cutaneous Invasive Aspergillosis

**TABLE 1 (Continued):**
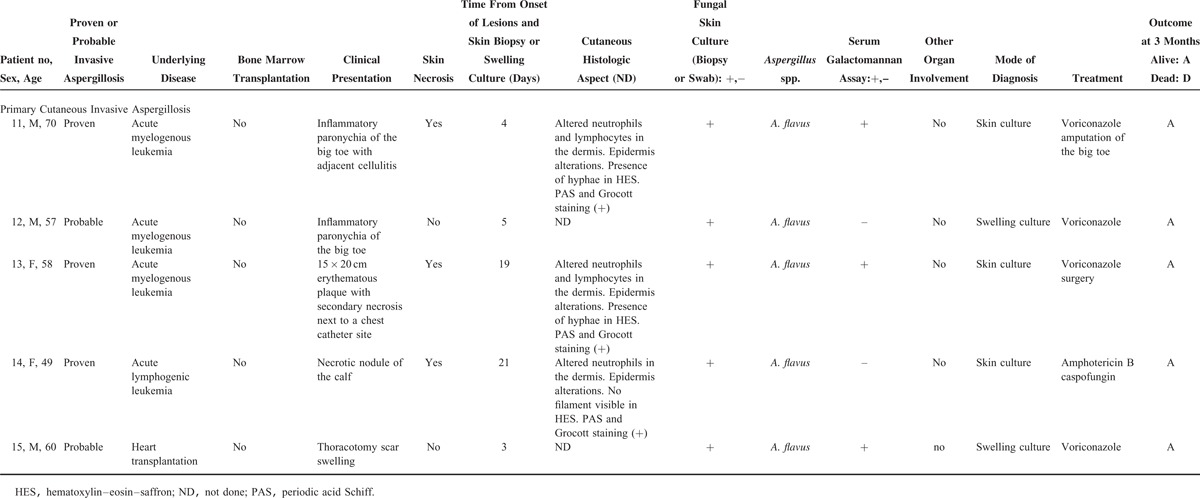
Clinical and Mycological Data for 15 Patients with Proven or Probable Cutaneous Invasive Aspergillosis

#### Dermatological Findings

**FIGURE 2:**
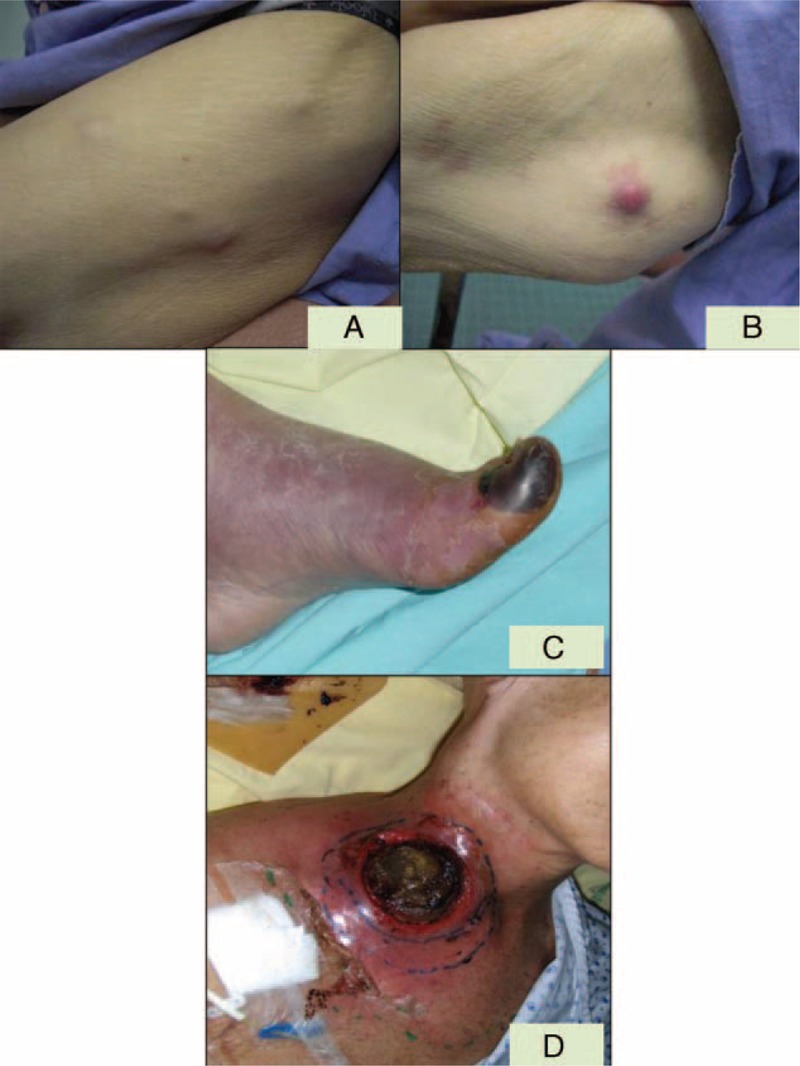
A and B, patient 4 with secondary CIA presenting as multiple nodules. C, patient 12 with primary CIA presenting as necrotic paronychia of the big toe. D, patient 14 with primary CIA presenting as a necrotic plaque on the chest. CIA, cutaneous invasive aspergillosis.

In SCIA, lesions were reported as unique or multiple papules or nodules in various localizations (Figure 2A and B). Lesions were described as erythematous, infiltrated, and inflammatory in every patient, and necrosis was observed in 2/10 patients. One patient had a periorbital cellulitis overlying a fungal sinusitis.

In PCIA, lesions presented as paronychia of the big toe in patients 11 and 12, with secondary necrosis and adjacent cellulitis of the leg in patient 11 (Figure 2C); patient 13 presented necrotic infiltrated plaque located next to a catheter insertion site (Figure 2D), patient 14 a necrotic nodule of the calf, and patient 15 swelling of a thoracotomy scar without mediastinitis (Table 1 ).

#### Mycological Findings

Mycological examination of cutaneous samples was performed on a skin biopsy in 13 patients and on a swab of purulent material in 2 (probable cases, patients 12 and 15). *Aspergillus* sp. grew from all skin biopsies of SCIA and represented *A. fumigatus* (6/10), *A. flavus* (3/10), and *A. nidulans* (1/10). *Aspergillus flavus* was the only species isolated in all PCIA cases. Serum galactomannan assay results were positive in 7/10 SCIA and 3/5 PCIA cases, and negative in other cases (cutoff for positivity 0.5).

#### Histologic Findings

**FIGURE 3:**
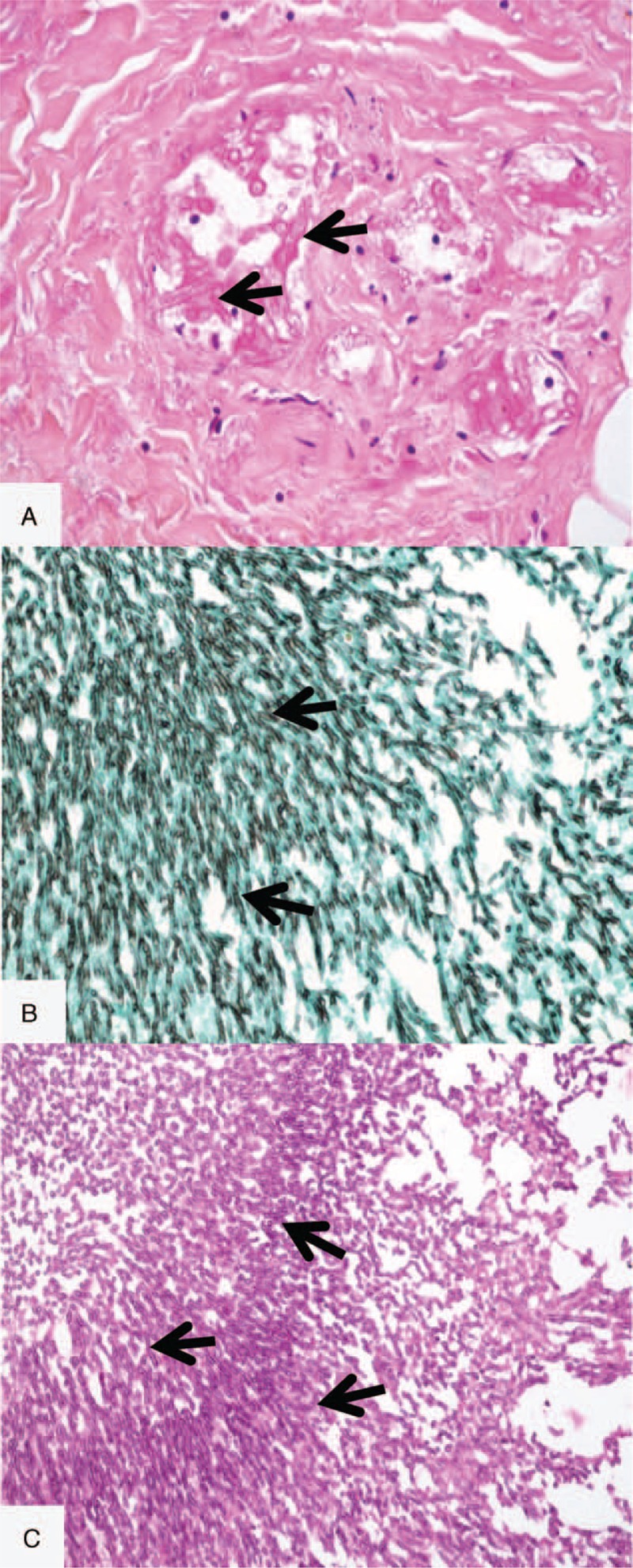
A, histologic findings of secondary CIA skin biopsy showing hyphae accumulated within small dermal necrotic vessels and surrounded by a few inflammatory cells (arrows, hematoxylin–eosin–saffron staining, 400 × original magnification). B, acute septate hyphae seen on Grocott staining (arrows, 200 × original magnification). C, periodic acid Schiff staining (arrows, 100 × original magnification). CIA, cutaneous invasive aspergillosis.

Histologic examination was performed in 11 patients (8 SCIA and 3 PCIA). In 9 biopsies (6 SCIA and 3 PCIA), the dermis was occupied by a polymorphic inflammatory infiltrate containing altered neutrophils (9/9), lymphocytes (5/9), and histiocytes (8/9) without plasma cells or granuloma formation. Small dermal vessels were often necrotic (Figure 3A). Two other biopsies (SCIA) were not contributive, showing nonspecific and mild inflammatory changes in the dermis and no fungal element. The epidermis was normal in SCIA and necrotic in 3/3 PCIA cases. In all 9 informative biopsies, acute septate hyphae were seen within or outside vessels on staining by hematoxylin–eosin–saffron (HES), Gomori-Grocott methenamine silver nitrate (Figure 3B), and periodic acid Schiff (PAS) (Figure 3C), but its detection was easier with Grocott than PAS staining in 3/9 cases. The fungus was visible in the dermis and hypodermis and classified as ramificated (5/9), septate (5/9), short (3/9), or with long hyphae (5/9). Hyphae grouped in clusters were observed in 2 SCIA patients.

#### Treatment and Follow-up

Antifungal therapy was initiated based on skin culture findings in 6/10 SCIA cases. Treatment for SCIA included voriconazole (5/10), amphotericin B (1/10), voriconazole and caspofungin (2/10), and amphotericin B and caspofungin (2/10). For PCIA, antifungal therapy consisted of voriconazole (4/5) or amphotericin B and caspofungin (1/5) and was associated with surgical detersion of necrosis in 2 patients.

Three months after the diagnosis of CIA, 7/10 SCIA patients (70%) had died, and all patients with PCIA were alive.

#### Literature Review

Five series of cutaneous IA cases (49 patients; 35 SCIA, 14 PCIA, Table 2) were published as of July 2014. The largest series was described in immunocompromised children.^11^ In adults, PCIA occurred on skin burns in 5 nonimmunocompromised patients and in immunosuppressed patients in the other cases. A clinical description was available for 9/35 SCIA cases and included necrotic papules, disseminated nodules or papules, or skin induration contiguous to an aspergillosis sinusitis. In total, 7/14 PCIA cases were described as necrotic or escharotic plaques. Histologic features of IA were provided for 18 patients (2 studies)^12,13^ and described as branched septate hyphae (14/18) or granuloma (4/18). *A. flavus* was predominantly isolated from PCIA patients in 1 series^12^ as in our study and *A. fumigatus* from SCIA patients. Positive serum galactomamann assay findings were not available in most previously published studies. As in our study, the prognosis was better with PCIA than SCIA (53% death rate in Burgos SCIA series).^11^

**TABLE 2:**
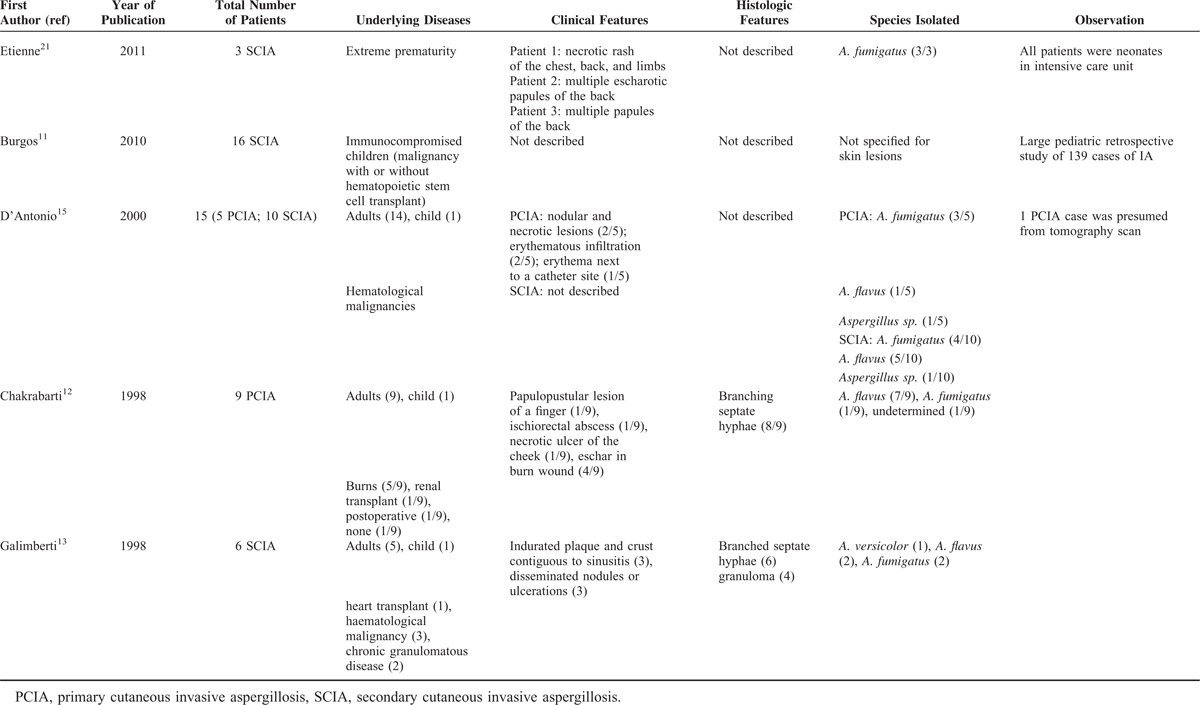
Review of Published Series of Cutaneous Aspergillosis

## DISCUSSION

The prevalence of cutaneous involvement in IA remained unknown to date. Patterson et al^14^ and D’Antonio et al^15^ estimated cutaneous involvement at 5% and 4% of IA, respectively, on the basis of retrospective studies.^,^ In our series, we could more precisely estimate the prevalence of cutaneous involvement in IA as at least 1% of 1410 cases of proven or probable IA. Most of our patients had an underlying hematological malignancy (80%), which is consistent with other studies.^11,16^ Secondary CIA has been reported in approximately 35 patients, in 2 pediatric series^11,21^ and 3 series of adults.^12,13,15^ In contrast, reports of PCIA in burn victims and immunocompromised or immunocompetent adults and children are numerous but isolated.^17,18^

The clinical features of our SCIA patients agreed with the previously reported description of SCIA.^9,19^ Thus, erythematous, disseminated papular, or nodular lesions should be suggestive of CIA in immunocompromised patients with hematological malignancies or solid organ transplantation and should prompt the clinician to immediately take a skin biopsy for histologic examination and mycological microscopy examination and culture. In our series, 2 patients with SCIA had necrotic nodules. Secondary CIA, however, may have a polymorphic presentation such as necrotic lesions or orbital cellulitis, usually more suggestive of mucormycosis and similar to the previously described erythema contiguous to aspergillosis sinusitis,^12^ or a unique lesion, which was not previously published and makes the diagnosis of dissemination when skin is the first site evidenced more challenging.

Primary cutaneous IA usually presents as isolated infiltrated lesions.^11,12,15,17^ In our series, 1 patient had an infiltrated plaque of the chest and 1 patient a nodule of the calf. We also observed atypical presentations such as 1 case of thoracotomy scar swelling without mediastinitis and 2 patients of necrotic paronychiae. Thus, we emphasize the need to evoke CIA with swelling of any localization in patients with hematological malignancy or solid organ transplantation. Furthermore, although predominant in previously published studies, secondary necrosis was observed in only 3/5 PCIA and 2/10 SCIA cases. We did not observe pustules, which are often reported in pediatric cases.^17^ A disruption of local skin integrity, often reported as a risk factor for PCIA,^18^ was observed in only 1 patient, with a large necrotizing lesion of the chest close to a catheter site. Pathophysiology of PCIA could result from direct inoculation from the environment through a central catheter, on necrotic skin lesions such as burns, or secondary dissemination from onychomycosis caused by *Aspergillus* infection in immunocompromised patients.

Histologic examination of skin biopsies mainly showed suggestive signs of infection with nonspecific, dermal, and nongranulomatous infiltrates with altered neutrophils and necrosis. We did not observe plasma cells or granuloma formations, in contrast with previous series of SCIA,^13^ and no adipocyte necrosis, in contrast with recent findings.^20^ The only difference between SCIA and PCIA was the presence of epidermal dystrophy or necrosis in PCIA patients, whereas the epidermis was normal in SCIA. Moreover, the morphologic features of fungal hyphae varied greatly, which could be because of variable times between antifungal treatment initiation and the skin biopsy. In detecting the fungus, sensitivity was higher for PAS and Gomori-Grocott methenamine nitrate silver than HES staining.

Fungal culture of a skin biopsy (or swab in 2 patients) allowed early diagnosis when performed in all patients, despite negative histologic findings in 2 patients. Sensitivity was greater for culture than microscopy of the fungus. In our study, biopsy was often delayed after onset of cutaneous symptoms, mostly in secondary forms, which explains why skin involvement allowed for diagnosing IA in 6/10 SCIA cases only. Misdiagnosis of CIA semiology may explain the delay in performing skin biopsy.

Most reported cases of PCIA and SCIA are caused by *A. flavus* and *A. fumigatus*, respectively,^8,11,20,21^ which agrees with our results. Of note, as was previously reported,^12,22,23^ only *A. flavus* was cultured from PCIA samples. Previously published studies showed that *A. flavus* and *A. fumigatus* have different pathogenicity pathways and are associated with different clinical presentation.^24^ Similarly, the preponderance of *A. flavus* has been described in the air of some tropical countries,^25^ but the country of origin was not captured in the case report form used here.

Positive serum galactomannan assay findings (7/10 SCIA and 3/5 PCIA) were not helpful in discriminating SCIA and PCIA, and positivity in SCIA cases was not associated with the extent of skin lesions.

The Infectious Disease Society of America has published guidelines for the treatment of IA.^26^ In accordance with these recommendations, voriconazole was the main antifungal agent used for first-line treatment in our study. Surgery is recommended for unique lesions of IA, but all of our patients presenting PCIA showed excellent 3-month prognosis, with surgical debridement performed in only 2.

In conclusion, our series of IA, although retrospective, is unique because it was extracted from a large prospective, exhaustive, and multicenter collection of IA cases. Primary cutaneous IA should be recognized early because the mycological features, management, and prognosis differ from those for SCIA, for which prognosis remains poor and is related to vascular invasion mostly in lungs and brain. In addition, the presence of any suppurative, necrotic, papulonodular, or infiltrated skin lesion in an immunocompromised patient should lead to immediate biopsy for histologic analysis and mycological skin direct examination and culture, to early diagnose a potential IA.
